# Anxiety and Depression in Patients with Traumatic Spinal Cord Injury: A Nationwide Population-Based Cohort Study

**DOI:** 10.1371/journal.pone.0169623

**Published:** 2017-01-12

**Authors:** Sher-Wei Lim, Yow-Ling Shiue, Chung-Han Ho, Shou-Chun Yu, Pei-Hsin Kao, Jhi-Joung Wang, Jinn-Rung Kuo

**Affiliations:** 1 Department of Neurosurgery, Chi-Mei Medical Center, Chiali, Tainan, Taiwan; 2 Institute of Biomedical Sciences, National Sun Yat-sen University, Kaohsiung, Taiwan; 3 Department of Nursing, Min-Hwei College of Health Care Management, Tainan, Taiwan; 4 Department of Medical Research, Chi-Mei Medical Center, Tainan, Taiwan; 5 Department of Pharmacy, Chia Nan University of Pharmacy and Science, Tainan, Taiwan; 6 Department of Medical Research, Chi-Mei Medical Center, Chiali, Tainan, Taiwan; 7 Department of Psychiatry, Chi-Mei Medical Center, Tainan, Taiwan; 8 Department of Neurosurgery, Chi-Mei Medical Center, Tainan, Taiwan; 9 Department of Biotechnology, Southern Taiwan University of Science and Technology, Tainan, Taiwan; Temple University School of Medicine, UNITED STATES

## Abstract

**Background:**

Traumatic spinal cord injury (tSCI) may involve new-onset anxiety and depression post-discharge. However, long-term population-based studies have lacked access to follow-up conditions in terms of new-onset anxiety and depression. The objective of this study was to estimate the long-term risk of new-onset anxiety and depression post-discharge.

**Methods:**

The Longitudinal Health Insurance Database 2000 (LHID2000) from Taiwan’s National Health Insurance Research Database was used in this study. Individuals with tSCI were identified using the International Classification of Diseases, Ninth Revision, Clinical Modification (ICD-9-CM) diagnostic codes of 806 and 952 from 1999–2008. The comparison cohort (other health conditions group) was randomly selected from the LHID2000 and was 1:1 matched by age, sex, index year, and comorbidities to reduce the selection bias. All study participants were retrospectively followed for a maximum of 3 years until the end of follow-up, death, or new-onset anxiety (ICD-9-CM: 309.2–309.4) or depression (ICD-9-CM: 296.2, 296.5, 296.82, 300.4, 309.0–309.1, and 311). Persons who were issued a catastrophic illness card for tSCI were categorized as having a severe level of SCI (Injury Severity Score [ISS] ≥16). Poisson regression was used to estimate the incidence rate ratios of anxiety or depression between patients with tSCI and other health conditions. The relative risk of anxiety or depression was estimated using a Cox regression analysis, which was adjusted for potential confounding factors.

**Results:**

Univariate analyses showed that the tSCI patients (n = 3556) had a 1.33 times greater incidence of new-onset anxiety or depression (95% confidence interval [CI]: 1.12–1.57) compared to the other health conditions group (n = 3556). After adjusting for potential risk factors, the tSCI patients had a significant 1.29-fold increased risk of anxiety or depression compared to the group with other health conditions (95% CI: 1.09–1.53). Individuals with tSCI, including patients who were under the age of 35, patients who were males, patients who had a low income, and patients without a Charlson Comorbidity Index score, all had a higher long-term risk of anxiety or depression than the other health conditions group (IRRs: 1.84, 1.63, 1.29, and 1.39, respectively). For all tSCI patients, those with an Injury Severity Score (ISS) ≥16 had an almost 2-fold higher risk of anxiety or depression (adjusted Hazard Ratio: 1.85; 95% CI: 1.17–2.92) compared to those with ISS <16.

**Conclusions:**

Our findings indicated that tSCI patients have a high risk of anxiety or depression post-discharge, especially among the younger tSCI patients (age <50 years), compared with the other health conditions group. This information could help physicians understand the long-term risk of new-onset anxiety or depression in tSCI patients post-discharge.

## Introduction

Spinal cord injury (SCI) is a devastating condition that may occur acutely (traumatic accidents such as traffic crashes, falls, sports injuries, or workplace accidents) [1–2] or chronically (illness such as spinal tumors or transverse myelitis) [3]. Globally, the highest prevalence of SCI is 906 per million in the US [4]. The injury usually results in symptoms of pain, disability, loss of function, and neurologic dysfunction [5,6], such as paralysis of voluntary muscles and loss of sensation below the level of the lesion, which is related to reduce mobility and functional independence. Additionally, people with SCI can be prone to complications such as pneumonia, septicemia, urinary tract infections, cardiac diseases and chronic pain, which may increase the clinical severity of their medical conditions [7, 8]. This might lower their quality of life in comparison with general population. [9]

In addition to physiological injuries, psychological disorders may occur after SCI. To estimate the prevalence of mood disorders in adults with tSCI, some studies have performed meta-analyses or systematic reviews about anxiety or depression following SCI. Le and Dorstyn [10] indicated that the prevalence of anxiety for self-reported measure estimates varied from 15–32%. Williams and Murry [3] estimated the prevalence of depression to be approximately 18.7% to 26.3%.

With respect to a population-based database in longitudinal studies, more self-reported and interview-based studies have been conducted. However, the timing of the assessment of psychological conditions after SCI could be viewed as a dynamic and individualized process [11]. SCI patients might experience chronic and lasting anxiety or depression in response to their injuries, and further understanding about the psychological disorders following SCI in the long term is necessary.

Craig et al. [12] performed a systematic review and revealed that SCI patients in the rehabilitation phase still have a risk of depression and that almost 30% of those may develop a risk of depression. In addition, the potential depressive risk may be approximately 27% when SCI patients return to their normal life. They suggested conducting a study with a heterogeneous sample of people with SCI from the acute injury stage through discharge and up to at least 2 years after the injury and indicated the importance of psychological morbidity after SCI. Craig et al. [13] conducted a longitudinal assessment of psychological disorders after SCI up to 6 months post-discharge. They found rates of psychological disorders of 17% to 25%, which were higher than those found in the Australian community. The study concluded that SCI could have a negative influence on mental health after 6 months post-discharge, and suggested that more studies should be conducted to address SCI and the transition into the community.

Arango-Lasprilla et al. [14] examined depression in cases with SCI during the first 5 years after their injuries. The prevalence of depression ranged from 11.9% at 1 year to 9.7% at 5 years post-SCI. The possible factors that influenced depression development included demographic characteristics, injury causes, and rehabilitation discharge factors. Kennedy and Roger [15] conducted a longitudinal study investigating 104 patients with SCI. The Beck Depression Inventory, Beck Hopelessness Scale, State Anxiety Inventory, and Social Support Questionnaire were used. Their results demonstrated a significant correlation between anxiety and depression in individuals with SCI. Furthermore, the longitudinal results provided an indicator of subtle changes in anxiety and depression over time.

There are few longitudinal population-based studies that have investigated the incidence of new-onset anxiety or depression after tSCI. Thus, in this study, we focused on tSCI and conducted a large nationwide Taiwanese population-based study to retrospectively examine the relationship between tSCI and the risk of new-onset anxiety or depression by following up at 3 years post-discharge in comparison with the other health conditions group. Anxiety and depression are common mental disorders and increasingly so in the Asia Pacific areas or Western countries [16, 17], and the genetic and environmental factors may also affect the development of anxiety and depression disorders [18]. In addition to their overlapping symptomatology and clinical presentation, they are highly comorbid with each other [19]. Furthermore, anxiety and depression are found in patients with chronic medical illnesses [20]. The combination of anxiety and depression may increase comorbidities, disabilities, the impact on quality of life, and healthcare utilization [19–21]. We sought to examine the contributory role of certain predisposing factors, including sociodemographic variables and the severity of tSCI.

## Materials and Methods

### Database

The Longitudinal Health Insurance Database 2000 (LHID2000) from January 1, 1997 to December 31, 2011 was used for this retrospective cohort study. LHID2000 is the subset database of Taiwan’s National Health Insurance Research Database (NHIRD), and the NHIRD was provided by the Bureau of National Health Insurance (BNHI) in Taiwan and contained 99% of inpatient and outpatient medical benefits participants from the Taiwanese population of 23 million individuals. The LHID2000 included 1 million beneficiaries randomly selected in 2000 from the NHIRD for research purposes.

### Ethics statement

For protecting patient’s privacy, encrypted personal identifications were used to prevent the possibility of an ethical violation according to regulations of the Bureau of National Health Insurance (BNHI) in Taiwan. The anonymous identification numbers with the linked claims information, such as sex, date of birth, medical services received, and prescriptions, can be used by the researchers. Therefore, informed consent was not required, and this study was approved for exemption by the Institutional Review Board (IRB) of Chi Mei Medical Center (IRB: 10307-E01). The IRB also specifically waived the consent requirement.

### Definition of individuals with tSCI

Patients with tSCI were selected from outpatient or inpatient claims. According to the International Classification of Diseases, Ninth Revision, Clinically Modified (ICD-9-CM) codes, the tSCI group was defined as those with a fracture of the vertebral column with tSCI (ICD-9-CM code: 806) or a spinal cord injury without evidence of spinal bone injury (ICD-9-CM code: 952).

Originally, the level of tSCI, such as tetraplegia, paraplegia or as American Spinal Injury Association (ASIA) levels, was not recorded in the medical records used for our study. As a proxy, the Injury Severity Score (ISS) was applied. The ISS is an anatomical scoring system that provides an overall score for patients with multiple injuries. Each injury is assigned an Abbreviated Injury Scale (AIS) score and is allocated to one of six body regions (head, face, chest, abdomen, extremities, and external). The AIS is an anatomically based consensus-derived global severity scoring system that classifies every injury in each body region according to its relative severity on a six-point ordinal scale (1: minor, 2: moderate, 3: serious, 4: severe, 5: critical, and 6: maximal, currently untreatable). Only the highest AIS score in each body region is used, and the 3 most severely injured body regions have their score squared and added together to produce the ISS score, which range from 1 to 75 [22].

In Taiwan, to decrease the financial burden of patients with certain serious illnesses, patients are issued a catastrophic illness card by the BNHI. The application of a catastrophic illness card must be signed by a certificated physician after the diagnosis and numbers of visits are verified. Therefore, individuals who had been issued a catastrophic illness card for SCI were categorized as having a severe level of SCI (Injury Severity Score [ISS] ≥16; patients with ISS ≥16 are defined as catastrophic injury in Taiwan) by the BNHI [23].

### Study population

The study group (newly diagnosed individuals with tSCI [tSCI group]) and the comparison group (individuals with other health conditions but without tSCI [other health conditions group]) were selected from the LHID2000 between January 1, 1999, and December 31, 2008. All participants were adults and aged ≧18 years. To ensure that all study participants had their first hospitalization for tSCI, we excluded patients who had a history of tSCI in 1997–1998. Those with missing data on sex were also excluded.

The other health conditions group was selected from the remaining patients without any diagnosis records of tSCI from the LHID2000, which was 1:1 matched for age, sex, comorbidities (hypertension, diabetes, and coronary heart disease), and index year within the same period. The tSCI group and other health conditions group were retrospectively followed for a maximum of 3 years until the end of follow-up, death, or a new-onset outcome. Individuals with a history of anxiety or depression prior to SCI or the index date for other health conditions group were excluded from the study.

### Definition of new-onset anxiety or depression disorder

Patients with anxiety or depression were identified either from outpatient claims or from inpatient hospitalization claims using the ICD-9-CM diagnosis codes during 1999–2011. The ICD-9-CM codes for anxiety, including anxiety, dissociative, and somatoform disorders (300 and 300.4 in the depression category) were as follows: predominant disturbance of other emotions (309.2), adjustment disorder with disturbance of conduct (309.3), and adjustment disorder with mixed disturbance of emotions and conduct (309.4). The ICD-9-CM codes for depression were as follows: major depressive disorder, single episode (296.2); bipolar I disorder, most recent episode (or current) depressed (296.5); depressive disorder, not elsewhere classified (311); dysthymic disorder (300.4); atypical depressive disorder (296.82); adjustment disorder with depressed mood (309.0); and prolonged depressive reaction (309.1). To verify the diagnosis of anxiety or depression, these codes was counted if the condition occurred either in inpatient claims or in at least three outpatient claims within one year.

In addition, to investigate the new-onset anxiety or depression after tSCI post-discharge, patients with anxiety or depression within the hospitalization period were excluded. To avoid other confounding factors which may affect anxiety or depression, the maximum follow-up time was 3-years after the index date. Moreover, the Charlson Comorbidity Index (CCI) score was used to estimate each individuals’ possible disease severity level [24, 25].

### Statistical analysis

All data processing and statistical analyses were performed with SAS 9.4 (SAS Institute Inc., Cary, NC, USA). Pearson’s chi-square test was used to analyze the difference between the tSCI group and the other health conditions group for categorical variables, including age, sex, economic status, medical comorbidities, and outcome. Age was categorized as follows: 18–35, 36–50, 51–65, 66–80, and >80 years. The economic status based on personal monthly income was classified into one of the following three categories: <$640 (new Taiwanese dollars [NTD] 1–19,999), $640–1,280 (NTD 20,000–39,999), or >$1,280 (NTD>40,000) [26]. Student’s t-test and the Wilcoxon rank-sum test were used to compare the time to anxiety or depression, respectively.

The incidence rate of anxiety or depression was calculated as the number of patients with anxiety or depression divided by the total number of person-years. To estimate the incidence rate ratios (IRRs) of anxiety or depression and the 95% confidence intervals (CI) among patients with tSCI, Poisson regression with total person-years as an offset variable was used. To present the proportion of patients who remained anxiety- or depression-free, the Kaplan-Meier curves were estimated, and a log-rank test was used to compare the difference between the tSCI and other health conditions groups. The relative risk of anxiety or depression was estimated by Cox regression analysis, which was adjusted for potential confounding variables such as age, sex, Charlson Comorbidity Index (CCI) score, economic status, and comorbidities (hypertension, diabetes, and coronary heart disease). The Kaplan-Meier curves were plotted using Stata 12 (Stata Corp., College Station, TX, USA). The significance level was set at p < .05. The estimation of the detectable hazard ratio difference was 1.08 according to a statistical power of 0.9 and a type I error probability of 0.05.

## Results

A total of 3,556 patients in the study group had a history of tSCI from 1999 to 2008, and the other health conditions group had 3,556 patients without any tSCI records during the study period. Those two groups were 1:1 matched by age, sex, and comorbidities (hypertension, diabetes, and coronary heart disease). The most common diagnosis for the group with other health conditions was acute upper respiratory infections of multiple or unspecified sites (ICD-9-CM: 465, approximately 9.00%).

Table 1 shows the baseline characteristics between the tSCI group and the other health conditions group. The economic status and Charlson Comorbidity Index (CCI) score presented significant differences between the tSCI group and other health conditions group. The outcome of anxiety or depression was different between the tSCI group and the other health conditions group (8.80% vs. 6.75%, respectively) during the study period. The tSCI group developed new-onset anxiety or depression (median: 1.08 years, interquartile range [IQR]: 0.36–1.96) earlier than the other health conditions group (median: 1.48 years, IQR: 0.70–2.23). In addition, 4.02% of the tSCI group had a higher level of injury (ISS ≥16).

**Table 1:** Sociodemographic characteristics and comorbidities in the tSCI group and other health conditions group.

	Other health conditions group	tSCI group	p-value^*^
	(N = 3,556)	(N = 3,556)	
Age group, n (%)			
18–35	925 (26.01)	926 (26.04)	1.0000
36–50	924 (25.98)	923 (25.96)	
51–65	759 (21.34)	760 (21.37)	
66–80	750 (21.09)	750 (21.09)	
>80	198 (5.57)	197 (5.54)	
Sex, n (%)			
Female	1,906 (53.60)	1,906 (53.60)	1.0000
Male	1,650 (46.40)	1,650 (46.40)	
Economic status, n (%)			
<$640	1,698 (47.75)	1,788 (50.28)	.0003
$640–1,280	1,359 (38.22)	1,379 (38.78)	
>$1,281	499 (14.03)	389 (10.94)	
Comorbidity, n (%)			
CCI score			
0	2,987 (84.00)	2,846 (80.03)	< .0001
1	367 (10.32)	419 (11.78)	
≥2	202 (5.68)	291 (8.18)	
HTN			
Yes	339 (9.53)	339 (9.53)	1.0000
No	3,217 (90.47)	3,217 (90.47)	
DM			
Yes	91 (2.56)	91 (2.56)	1.0000
No	3,465 (97.44)	3,465 (97.44)	
CAD			
Yes	51 (1.43)	51 (1.43)	1.0000
No	3,505 (98.57)	3,505 (98.57)	
Outcome information			
Time to anxiety/depression, yearsmedian (IQR)	1.48 (0.70–2.23)	1.08 (0.36–1.96)	.0007
Anxiety/depression, n (%)			
Yes	240 (6.75)	313 (8.80)	.0012
No	3,316 (93.25)	3, 243 (91.20)	
Type of anxiety/depression, n (%)			
Anxiety only	182 (5.12)	204 (5.74)	.0014
Depression only	50 (1.41)	105 (2.95)	
Anxiety and depression	8 (0.22)	4 (0.11)	
Level of SCI, n (%)			
ISS ≥16		143 (4.02)	
ISS <16		3,413 (95.98)	

^*^ The p-value is calculated from Student’s t-test for continuous variables and Pearson’s chi-squire test for categorical variables.

SCI, spinal cord injury; SD, standard deviation; CCI, Charlson Comorbidity Index; HTN, hypertension; DM, diabetes mellitus; CAD, coronary artery disease.

Table 2 compares the incidence rate of new-onset anxiety or depression between the patients with tSCI and the other health conditions group for overall and stratified analysis. The tSCI group had a 1.33 times greater incidence of new-onset anxiety or depression compared to the other health conditions group (Table 2). The incidence of anxiety or depression in tSCI patients increased from 181.51 to 501.21 among those aged 18–35 to >80. The incidence of new-onset anxiety or depression was significantly different in persons younger than 35 years (IRR: 1.84, 95% CI: 1.15–2.94). Patients with tSCI aged 36–50 also had a 1.43-fold greater risk of anxiety or depression compared with the other health conditions group (IRR: 1.43, 95% CI: 1.01–2.02).

**Table 2:** Incidence rate for anxiety or depression between the tSCI group and other health conditions group.

	Other health conditions group (N = 3,556)			tSCI group (N = 3,556)			IRR (95% CI)	p-value	Adjusted^**^ HR (95% CI)	p-value
	Anxiety/depression	PY	Rate*	Anxiety/depression	PY	Rate*				
Total	240	10,300.40	233.00	313	10,105.28	309.74	1.33 (1.12–1.57)	0.0009	1.29 (1.09–1.53)	.0030
Level of SCI										
ISS <16				292	9,709.09	300.75				
ISS ≥16				21	396.20	530.04				
Age										
18–35	27	2,733.98	98.76	49	2,699.61	181.51	1.84 (1.15–2.94)	0.0111	1.00 (ref.)	
36–50	54	2,694.04	200.44	75	2,623.55	285.87	1.43 (1.01–2.02)	0.0467	1.74 (1.31–2.31)	.0001
51–65	68	2,155.77	315.43	70	2,156.93	324.54	1.03 (0.74–1.44)	0.8673	2.01 (1.51–2.67)	< .0001
66–80	73	2,142.83	340.67	92	2,086.51	440.93	1.29 (0.95–1.76)	0.0998	2.14 (1.60–2.87)	< .0001
>80	18	573.78	313.71	27	538.70	501.21	1.60 (0.88–2.90)	0.1236	2.10 (1.42–3.10)	.0002
Sex										
Male	76	4,833.55	157.23	121	4,729.31	255.85	1.63 (1.22–2.17)	0.0009	1.00 (ref.)	
Female	164	5,466.85	299.99	192	5,375.97	357.15	1.19 (0.97–1.47)	0.1010	1.48 (1.24–1.76)	< .0001
Economic status										
<$640	138	4,884.50	282.53	184	5,044.61	364.75	1.29 (1.04–1.61)	0.0233	1.93 (1.32–2.81)	.0007
$640–1,280	86	3,945.46	217.97	113	3,919.38	288.31	1.32 (0.99–1.75)	0.0507	1.70 (1.16–2.48)	.0062
>$1,280	16	1,470.45	108.81	16	1,141.30	140.19	1.29 (0.64–2.58)	0.4736	1.00 (ref.)	
Comorbidity										
HTN	31	970.18	319.53	36	956.63	376.32	1.18 (0.73–1.90)	0.5043	0.78 (0.58–1.05)	.1058
DM	7	264.47	264.69	11	251.37	437.60	1.65 (0.64–4.26)	0.2984	0.74 (0.44–1.22)	.2364
CAD	6	144.66	414.75	9	136.76	658.09	1.59 (0.56–4.46)	0.3811	1.48 (0.86–2.55)	.1625
CCI score										
0	173	8,703.11	198.78	225	8,137.91	276.48	1.39 (1.14–1.70)	0.0011	1.00 (ref.)	
1	41	1,041.73	393.57	54	1,152.81	468.42	1.19 (0.79–1.79)	0.4006	1.58 (1.25–2.01)	.0002
≥2	26	555.56	468.00	34	814.56	417.40	0.89 (0.54–1.49)	0.6605	1.53 (1.14–2.06)	.0048

PY, person-year; IRR, incidence rate ratio; CI, confidence interval; HR, hazard ratio; ref., reference; HTN, hypertension; DM, diabetes mellitus; CAD, coronary artery disease; CCI, Charlson Comorbidity Index.

*rate: per 10,000 person-years.

**The hazard ratio were adjusted by age, sex, economic status, comorbidities, and CCI score.

Women with tSCI had a higher incidence rate than men (357.15 per 10,000 person-years vs. 255.85 per 10,000 person-years). However, compared with the other health conditions group, men with tSCI had a higher ratio of new-onset anxiety or depression (IRR: 1.63, 95% CI: 1.22–2.17) than did women with tSCI (IRR: 1.19, 95% CI: 0.97–1.47).

Regarding the socioeconomic factors, patients with tSCI and a low economic status (income <$640) had a higher anxiety or depression incidence rate (364.75 per 10,000 person-years) with a significant IRR of anxiety or depression compared to the other health conditions (IRR: 1.29, 95% CI: 1.04–1.61). In addition, regarding comorbidities, among those without any comorbidity (CCI = 0), patients with tSCI had a 1.39-fold greater IRR (95% CI: 1.14–1.70) of anxiety or depression compared to those who had other health conditions.

**Fig 1:**
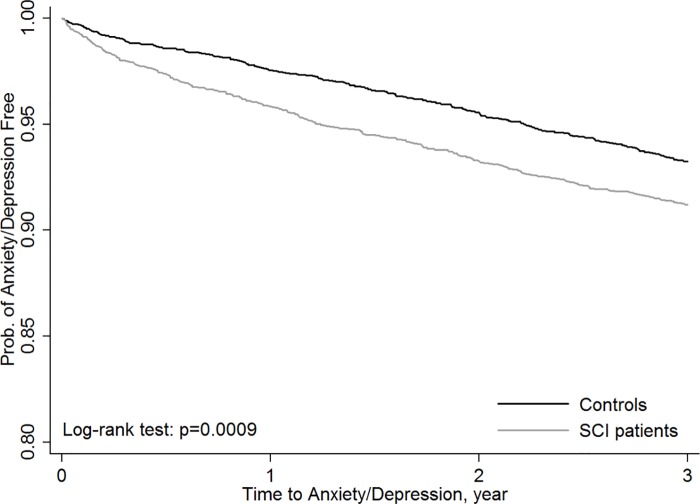
Kaplan-Meier plot for anxiety or depression occurrence for tSCI and the other health conditions groups. (a) anxiety/depression-free survival curves for patients with the spinal cord injury (tSCI patients, n = 3,556) and the other health conditions groups (n = 3,556) during the 3-year follow up period (p = .0009). (b) The significant level was set at p < .05. (c) It showed that the SCI patients had a significantly higher risk of new-onset anxiety or depression than the other health conditions group.

Considering the adjusted confounding factors, the Cox proportional hazard analysis showed that the tSCI group was more prone to develop anxiety or depression than the other health conditions group (adjusted HR: 1.29, 95% CI: 1.09–1.53) (Table 2). Additionally, the Kaplan-Meier plots showed that the tSCI group had a significantly higher risk of new-onset anxiety or depression than the other health conditions group (p = 0.0009) (Fig 1).

In this study, injuries were categorized into two levels of severity: ISS <16 and ISS ≥16. After adjusting for age, sex, economic status, and comorbidities, both levels of SCI severity in the tSCI group were associated with a higher risk of new-onset anxiety or depression than the other health conditions group (HR for ISS ≥16: 2.36, 95% CI: 1.50–3.72; HR for ISS <16: 1.25, 95% CI: 1.06–1.49). Furthermore, for the tSCI group only, those with ISS ≥16 had a 1.85-fold risk of new-onset anxiety or depression compared to those with tSCI and ISS <16 (95% CI: 1.17–2.92; Table 3).

**Table 3:** Cox model for the risk of anxiety or depression by the different levels of tSCI compared with other health conditions and tSCI only.

	Adjusted^*^ HR (95% CI)	p-value	Adjusted^*^ HR for tSCI only (95% CI)	p-value
Other health conditions	1.00 (ref.)			
tSCI group: ISS<16	1.25 (1.06–1.49)	.0101	1.00 (ref.)	
tSCI group: ISS≥16	2.36 (1.50–3.72)	.0002	1.85 (1.17–2.92)	.0082
Age				
18–35	1.00 (ref.)		1.00 (ref.)	
36–50	1.73 (1.30–2.30)	.0001	1.57 (1.09–2.25)	.0144
51–65	2.01 (1.51–2.68)	< .0001	1.61 (1.11–2.33)	.0121
66–80	2.17 (1.62–2.91)	< .0001	1.99 (1.36–2.90)	.0004
>80	2.13 (1.44–3.15)	.0001	2.21 (1.34–3.63)	.0018
Sex				
Male	1.00 (ref.)		1.00 (ref.)	
Female	1.51 (1.26–1.80)	< .0001	1.36 (1.08–1.73)	.0103
Economic status				
<$640	1.87 (1.28–2.73)	.0012	1.98 (1.17–3.36)	.0113
$640–1,280	1.68 (1.15–2.45)	.0074	1.79 (1.05–3.03)	.0320
>$1,281	1.00 (ref.)		1.00 (ref.)	
CCI score				
0	1.00 (ref.)		1.00 (ref.)	
1	1.59 (1.25–2.01)	.0001	1.47 (1.07–2.01)	.0177
≥2	1.51 (1.13–2.03)	.0060	1.23 (0.83–1.82)	.2978
HTN				
Yes	0.79 (0.59–1.06)	.1165	0.75 (0.50–1.12)	.1569
No	1.00 (ref.)		1.00 (ref.)	
DM				
Yes	0.72 (0.43–1.19)	.2012	0.91 (0.47–1.75)	.7682
No	1.00 (ref.)		1.00 (ref.)	
CAD				
Yes	1.49 (0.86–2.57)	.1524	1.69 (0.83–3.43)	.1492
No	1.00 (ref.)		1.00 (ref.)	

^*^ The adjusted HRs were adjusted by age, sex, economic status, comorbidities, and CCI score. CI, confidence interval; ref., reference; HR, hazard ratio; CCI, Charlson Comorbidity Index; HTN, hypertension; DM, diabetes mellitus; CAD, coronary artery disease.

## Discussion

To the best of our knowledge, this is the first study to use a large nationwide population-based cohort to analyze new-onset anxiety or depression after individuals with tSCI post-discharge compared to individuals with other health conditions in the long term. In general, most previous studies have used questionnaires for self-rating anxiety or depression [9, 13–15, 23]. In Taiwan, clinical professionals used questionnaires, such as the Hamilton Depression Rating Scale, HAM-D, Beck Depression Inventory, Hamilton Anxiety Scale, Beck Anxiety Inventory, and interviews to arrive at a diagnostic code. Therefore, this study used ICD-9-CM codes to define anxiety or depression.

Additionally, for estimating anxiety or depression after tSCI in the long-term, we excluded participants who experienced anxiety or depression followed by tSCI during the hospitalization period. Although our study only included individuals seeking a medical evaluation were identified by the ICD-9-CM which probably caused lower rates of anxiety and depression, the national database contains nearly 99% of the inpatient and outpatient medical beneficiaries of the 23 million Taiwanese residents; thus, the results of our analysis closely approximate the reality of anxiety or depression between patients with and without tSCI in Taiwan. This information is crucial to facilitate the prevention and treatment of tSCI-related dysfunctions.

Over this 3-year follow-up period, we found that tSCI was significantly related to the occurrence of new-onset anxiety or depression (1.33 times the rate among the other health conditions group). After adjusting for the participants’ sociodemographic data, including age, sex, economic status, and comorbidities, tSCI was associated with a significantly increased risk of anxiety or depression (HR: 1.29). Our results were consistent with those of Williams and Murray [3]. They performed a meta-analysis of screening studies on depression after SCI, which included the diagnostic tools that were used to measure depression, including unstructured, semi-structured, or structured clinical interviews and/or a clinician diagnosis. They concluded that the prevalence of depression after SCI is substantially greater than that of the general medical population. However, the anxiety or depression rate after SCI was 8.80% in our study; this rate seemed lower than in general studies, meta-analyses or systematic reviews [27, 28]. Saunders et al. [29] examined a large number (n = 801) of American adults with tSCI over a 5-year period who averaged 15 or more years post-injury. Participants responded to two questionnaires, one in 2002 (Time 1) and another in 2008 (Time 2). The proportion with clinically significant symptoms of depression was 41.4%, which was much higher than our results. This might be due to different measurements of psychological disorders (questionnaire-based vs. medical records) and participants with a different number of years post-injury. The participants in Saunders et al. averaged 15 or more years post-injury, and there might have been other confounders that influenced the occurrence of depression. Williams and Murray [3] revealed an estimation of depression diagnosis after SCI of 18.7%-26.3%. Craig [12] indicated that persons with SCI have a 30% risk of accompanying depression in the rehabilitation phase, approximately 27% in the community. Le and Dorstyn [10] assessed the anxiety prevalence using individual self-report measures and found rates ranging from 15% to 32%.

Although the incidence of anxiety or depression was higher in persons with tSCI, the leading causes are still not clear. Previous studies have suggested that tSCI can advance the deterioration of functions (e.g., sexual functioning, alterations in the urinary tract) and cause individuals to have negative expectations of the future [30]; in addition, tSCI-related pain has been associated with changes in cognition, including increased levels of depression, anxiety, anger, or psychosocial impairment [31]. These viewpoints may explain why individuals with tSCI exhibit anxiety or depression. Untreated depression is the number one cause of suicide, and suicide was the eleventh leading cause of death in Taiwan in 2014. [32] Thus, this issue merits further evaluation.

Furthermore, anxiety was proportionally higher in comparison to depression in patients with tSCI within the 3-year follow-up. One explanation is that anxiety occurred within the early stages of the injury as a mechanism of coping with the situation caused by tSCI.

Previous studies have evaluated the progress of depression after SCI [12, 31, 32]. Hoffman et al. [33] performed a longitudinal study that examined the change in rates of depression in patients with SCI from 1–5 years and found a small overall decrease in the prevalence of depression over time (21–18%). Dryden et al. [34] demonstrated that 34 of the examined 201 traumatic patients with SCI were diagnosed with depression and that among those with depression, 50% had recovered 6 years after SCI.

The two aforementioned studies focused on depression. Although fewer than those for depression, there were some studies that have examined anxiety or mental health over time. [11, 15] In the future, it will still be an important issue to compare the clinical improvement and to detect the duration and progress of both anxiety and depression over time for patients with tSCI. If anxiety occurs rapidly and more often after injury compared to depression within the early periods, perhaps anxiety will worsen gradually into chronic depression if treatment is not provided as soon as possible. This might remind clinicians that they have the responsibility to perceive psychiatric differences in patients with SCI and to treat them in a timely manner. Medication (e.g., antidepressants) and psychological interventions (e.g., cognitive behavioral therapy (CBT) and coping effectiveness training (CET)) have been targeted for the treatment of anxiety or depression post-tSCI.

Our results for age differed from those of previous studies. Cook [35] reported that age was significantly correlated with depression but not anxiety, and that those who were older (aged 35–67 years) had higher depression scores than younger individuals did (aged 15–34 years). However, Scivoletto [36] and Migliorini [37] and colleagues demonstrated that age was not associated with anxiety or depression.

The present study reported that men with tSCI had a 1.63-fold higher risk of anxiety or depression than the other health conditions group. In addition, men with tSCI would have a higher risk of anxiety or depression compared to men without tSCI. In our study, 73.38% of individuals with tSCI were aged ≤65 years and 46.40% were men. In traditional Taiwanese society, men, especially those of working age, are often responsible for earning the income. However, once men have tSCI, their self-esteem, confidence, and working abilities may negatively affect their physical functions, leading to unemployment. This would lead to frequent socioeconomic difficulties, causing men with tSCI to be more likely to develop psychological disorders or mental illness compared to the other health conditions group.

In addition, after controlling for other variables in the tSCI group, the lowest and median income levels (<$1,281, i.e., NTD <39,999) were associated with a higher risk of anxiety or depression compared to the high income level (≥$1,281, i.e., NTD ≥40,000) (adjusted HRs 1.93 and 1.70, respectively). Our study had the same conclusion as previous studies. Krause et al. [38] examined the mental health outcomes of individuals with tSCI at different income levels. Reportedly, a lower household income may increase the probability of poorer mental health.

To our knowledge, this is the first study to analyze the relationship between the severity of tSCI based on ISS ≧16 for persons who have been issued a catastrophic illness card for tSCI in Taiwan. It has been reported that a severe level of tSCI is associated with a higher risk of anxiety or depression, which was consistent with our findings. Thus, in the interest of the patient’s mental health, clinicians should pay more attention to tSCI patients.

### Strengths and limitations

Our study has several strengths. First, previous studies commonly used questionnaires, interview-based data, or small sample sizes to evaluate the relationship between SCI and anxiety or depression. By contrast, we used a large nationwide population-based database and adequate statistical power to demonstrate differences in the risks of tSCI related to anxiety or depression. In addition, we compared the persons with anxiety or depression after SCI to the other health conditions group. Second, our study was based on longitudinal claims, which helped minimize the potential for selection bias. We focused not on the acute stage, but rather on the long-term psychological situation in tSCI patients post-discharge. Third, we adjusted for age, sex, and comorbidities, which can have independent effects on the risk of anxiety or depression in patients with tSCI. Fourth, the risks of anxiety or depression with different severities of tSCI were studied and compared. The valid diagnosis of SCI and psychiatric disorders by neurosurgeons and psychiatrists were recorded in the claims database.

There are also some limitations in this study that need to be addressed. First, we did not have enough information from the claims database to determine the degree of anxiety or depression in patients with tSCI over time. Second, we could not indicate with certainty whether the diagnostic tools or validated scales for anxiety or depression were used in accordance with Diagnostic and Statistical Manual of Mental Disorders (DSM) criteria, nor could we distinguish the patients’ level of tSCI (such as tetraplegia, paraplegia or as ASIA levels). The study relied on coding ICD-9 CM in the claims, which may have caused a disease misclassification bias. However, the disease coding came from specialists, and the database was released from the BNHI in Taiwan, which made it a credible source to study. Third, because we focused on the impact of new-onset anxiety and depression after tSCI for the long-term in this study, we did not compare the Asian Pacific areas to Western countries concerning the differences in anxiety or depression after tSCI. In addition, we did not demonstrate variable mental health pathways at different time intervals from the onset of tSCI. These topics merit in-depth discussion in our future research. Fourth, in this study, we were limited to the database LHID2000. Thus, the other health conditions group included individuals who were all medical beneficiaries with other medical conditions except tSCI, which could have adversely influenced their mood and reduced the magnitude of differences in relative risk. Finally, the prevalence of anxiety or depression in our study may have been underestimated because only individuals seeking a medical evaluation could be identified.

## Conclusions

Individuals with tSCI have a higher risk of anxiety or depression than individuals in the other health conditions group. Thus, physicians and family members should pay attention to the patients’ or their relatives’ anxiety or depression. Our study highlights the importance of routine psychological assessment throughout the spinal rehabilitation process.

We hope that increasing awareness of the incidence and risk factors for new-onset anxiety or depression in patients with tSCI can improve clinicians’ understanding of the injuries, as well as the treatment and long-term rehabilitation protocols. Because of improved survival rates after tSCI [39], the extent and nature of any negative psychological effects associated with tSCI needs to be carefully determined. More social resources and/or government welfare policies should be invested in improving the mental health of adults with tSCI.
